# Transplantation of human Wharton’s jelly-derived mesenchymal stem cells highly expressing TGFβ receptors in a rabbit model of disc degeneration

**DOI:** 10.1186/s13287-015-0183-1

**Published:** 2015-10-02

**Authors:** Jongchan Ahn, Eun-mi Park, Byeong Ju Kim, Jin-Soo Kim, Bogyu Choi, Soo-Hong Lee, Inbo Han

**Affiliations:** Department of Biomedical Science, CHA University, 335 Pangyo-ro, Bundang-gu, Seongnam-si, Gyeonggi-do, 463-400 South Korea; Department of Neurosurgery, CHA University, CHA Bundang Medical Center, 59 Yatap-ro, Bundang-gu, Seongnam-si, Gyeonggi-do, 13496 South Korea

## Abstract

**Introduction:**

Mesenchymal stem cells (MSCs) are widely considered to hold promise for the treatment of intervertebral disc (IVD) degeneration. However, variation in the therapeutic efficacy of MSCs is a major problem and the derivation of MSCs for use in IVD regeneration has not been optimized. Additionally, no data are available on the efficacy of Wharton’s Jelly-derived MSC (WJ-MSC) transplantation in an animal model of IVD degeneration.

**Methods:**

This study evaluated the effectiveness of a cross-linked hyaluronic acid (XHA) scaffold loaded with human WJ-MSCs, according to their expression levels of transforming growth factor-β receptor I/activin-like kinase receptor 5 (TβRI/ALK5) and TβRII, for IVD regeneration in a rabbit model. We compared the degree of IVD regeneration between rabbits transplanted with a XHA scaffold loaded with WJ-MSCs highly and lowly expressing TβRI/ALK5 and TβRII (MSC-highTR and MSC-lowTR, respectively) using magnetic resonance imaging (MRI) and histological analysis.

**Results:**

At 12 weeks after transplantation, T2-weighted MRI analysis showed significant restoration of the disc water content in rabbits treated with a MSC-highTR-loaded XHA scaffold in comparison to rabbits treated with the scaffold alone or a MSC-lowTR-loaded XHA scaffold. In addition, morphological and histological analyses revealed that IVD regeneration was highest in rabbits transplanted with a MSC-highTR-loaded XHA scaffold.

**Conclusion:**

Taken together, our results suggest that a MSC-highTR-loaded XHA scaffold supports IVD regeneration more effectively than a MSC-lowTR-loaded XHA scaffold. This study supports the potential clinical use of MSC-highTR-loaded XHA scaffolds to halt IVD degeneration or to enhance IVD regeneration.

**Electronic supplementary material:**

The online version of this article (doi:10.1186/s13287-015-0183-1) contains supplementary material, which is available to authorized users.

## Introduction

Approximately 80 % of the population experience at least one episode of low back pain at some point during their lifetime and low back pain is a leading cause of adulthood disability [1]. Intervertebral disc (IVD) degeneration is considered to be a major cause of low back pain, even though the definite etiology of IVD degeneration is largely unknown [2–4]. In contrast to articular cartilage, the IVD has three components: the nucleus pulposus (NP), the annulus fibrosus (AF), and the cartilage endplate. IVD degeneration is characterized by the progressive loss of NP cells and extracellular matrix (ECM) components such as proteoglycan and collagen type II [5–9]. In general, conservative treatments for symptomatic IVD degeneration such as medications and physiotherapy are currently used as the first-line treatment to manage low back pain. When conservative treatments fail, surgical treatments including excision of the degenerated disc alone or in combination with a spinal fusion procedure may be indicated. Although symptoms frequently improve following these surgeries, the improvement is often temporary and the operated level of the spine, as well as levels adjacent to it, can exhibit accelerated degeneration that requires additional surgery [10, 11]. There is thus a strong clinical demand for the development of new biological approaches such as cell-based therapies to impede IVD degeneration and/or to regenerate the degenerated IVD in order to cure low back pain and maximize functional recovery.

In animal models of IVD degeneration, mesenchymal stem cells (MSCs) from different sources have shown promising results in regenerating degenerated IVD. The xenograft animal models have also been utilized for analysis of human MSCs and have produced a number of important successes [12–16]. However, there is no clear recommendation as to which type of human MSCs are most efficacious in this regard. Additionally, variation in the therapeutic efficacy of MSCs due to their different differentiation capacity is one of the major problems. For example, our previous study [17] reported that adipose tissue-derived MSCs (AD-MSCs) isolated from different donors exhibit different levels of chondrogenic differentiation. Furthermore, the expression levels of transforming growth factor-beta receptor I/activin-like kinase receptor 5 (TβRI/ALK5) and TβRII are directly linked with the ability of MSCs to undergo chondrogenesis [17–19]. With regards to improving the therapeutic potential of transplanted MSCs, a scaffolding technology is also considered to be important to prevent cell leakage and reduce the risk of uncontrolled MSC differentiation into osteoclasts leading to osteophyte formation. Indeed, rabbits exhibit osteophyte growth in the anterolateral disc space due to cell leakage after MSC transplantation [20, 21].

Wharton’s Jelly-derived MSCs (WJ-MSCs) have gained attraction as an alternative source of stem cells because of their ease of isolation, high expansion rate, hypoimmunogenicity, and unique immunomodulatory properties compared with other MSCs [22–26]. Although chondrogenic potential of WJ-MSCs has been described, no studies have shown the efficacy of WJ-MSC transplantation for IVD regeneration [12]. Thus, in the present study we investigated whether WJ-MSCs highly expressing TβRI/ALK5 and TβRII (MSC-highTR) are more effective for IVD regeneration than WJ-MSCs lowly expressing TβRI/ALK5 and TβRII (MSC-lowTR) in a rabbit model of IVD degeneration. Furthermore, we also evaluated the effectiveness of the combined use of WJ-MSCs and a cross-linked hyaluronic acid (XHA) scaffold for IVD regeneration.

## Materials and methods

### Isolation and culture of human WJ-MSCs

With the written consent of the parents and the approval (No. BD2013-007D) of the Ethics Committee of our institute, fresh human umbilical cords were obtained immediately after birth and collected in sterile boxes containing phosphate-buffered saline (PBS). WJ-MSCs were prepared as described elsewhere [22–26] and all culture conditions adhered to Good Manufacturing Practice (GMP) standards. Briefly, MSCs were isolated within 2 h of obtaining umbilical cords. The umbilical cords were disinfected in 75 % ethanol for 30 s and cleaned in PBS. The blood vessels were removed, and then Wharton’s jelly was peeled off from the remaining part of the umbilical cords. The Wharton’s jelly was transferred to a sterile container and washed thoroughly three times in serum-free Dulbecco’s modified Eagle’s medium (DMEM; Gibco BRL, Gaithersburg, MD, USA) containing penicillin 100 μg/ml, streptomycin μg/ml, and amphotericin B 250 μg/ml. The jelly was then cut into pieces smaller than 0.5 cm^3^. The minced Wharton’s jelly was digested for 6–10 h in a sterilized bottle with 15 ml culture medium containing collagenase of type I at 0.075 % in 5 % carbon dioxide, at 37 °C with agitation in an incubator. The cells were then washed three times with D-Hank’s salt solution and centrifuged at 250 × *g* for 10 min at room temperature, and the cells were resuspended in culture medium DMEM with low glucose (DMEM-L; Gibco BRL) supplemented with 10 % (v/v) fetal bovine serum (FBS; Gibco BRL) and 1 % antibiotic–antimycotic solution (Gibco BRL) in humidified air with 5 % carbon dioxide at 37 °C. Once the flask reached approximately 80 % confluence, the WJ-MSCs were subcultured using a seeding density of 5 × 10^3^ viable cells/cm^2^. It has been reported that WJ-MSCs, even in the first six or seven passages, seem to possess good quantitative and qualitative traits (clonal ability, etc.) [23]. Our data also showed that WJ-MSCs maintained high viability and pluripotency without any karyotype abnormalities until the nineteenth passage (data not shown). The seventh passage of WJ-MSCs was therefore used for our study.

### Examination of expression levels of TβRI/ALK5 and TβRII in WJ-MSCs using fluorescence-activated cell sorting analysis

We examined the expression of TβRI/ALK5 and TβRII in WJ-MSCs from four different donors by fluorescence-activated cell sorting (FACS) analysis using TβRI (ABF17; Millipore, Billerica, MA, USA) and TβRII (ab78419; Abcam, Cambridge, MA, USA) antibodies. Cultured cells from each of the four donors were collected by trypsinization and centrifuged at 300 × *g* for 10 min. The cells were washed three times with cold FACS buffer (1 % bovine serum albumin (BSA), 0.1 % sodium azide in PBS) and incubated for 1 h in PBS containing 3 % BSA for blocking, followed by 30 min of incubation with primary antibodies (1 μg/1 × 10^6^ cells). Cells were washed three times with cold FACS buffer and then incubated with second antibody (Alexa Fluor® 488, A-11008 or A-11001; Thermo Fisher Scientific, Hudson, NH, USA) at 1/500 dilution for 30 min. Isotype control antibodies were used under the same conditions. Cells were washed three times with cold FACS buffer and then analyzed using a FACS accuri C6 machine (BD Biosciences, Billerica, MA, USA).

### XHA scaffold for cell delivery: evaluation of viscosity and cytotoxicity

Tissuefill® (hyaluronic acid derivatives; CHA Meditech Co., Ltd, Daejeon, Korea) is a clear, transparent, and viscoelastic gel composed of hyaluronic acid, which is derived from nonanimal origins. The gel is cross-linked with butanediol diglycidyl ether, and is resolved almost completely in the body via enzymatic reactions. This XHA is approved by the South Korean Ministry of Food and Drug Safety and has been in common use for filling tissue defects. Tissuefill® was donated from CHA Meditech Co., Ltd to examine the effectiveness of combined use of WJ-MSCs and the XHA. To develop an injectable WJ-MSC-loaded XHA scaffold for IVD regeneration, we examined the optimal concentration of XHA and also evaluated whether the XHA induced WJ-MSC toxicity. To determine the concentration of XHA, 2, 1, 0.5 and 0.1 % XHA solutions were applied to the culture plate and then the shape of the XHA hydrogel solution was observed. After determining the optimal concentration of XHA, the cytotoxicity of the XHA was investigated using a Cell Counting Kit-8 (CCK-8; ) and the dual-fluorescence Live/Dead® cell viability assay (). Live cells were stained green with Calcein-AM and dead cells were stained red with Ethidium homodimer-1.

### Comparison of the chondrogenic potential of WJ-MSCs in vitro

In vitro, we compared chondrogenic potentials between MSC-lowTR and MSC-highTR. Western blotting was performed to compare the endogenous collagen type II expression between MSC-lowTR and MSC-highTR as described previously [16]. Briefly, WJ-MSCs were incubated with lysis solution from a commercially available RIPA buffer (R0278; Sigma-Aldrich, St. Louis, MO, USA). Equal amounts of cells were separated in a 12 % sodium dodecyl sulfate polyacrylamide gel and transferred onto polyvinylidene fluoride (PVDF) membranes using the cassette in the transfer tank (Bio-Rad, Hercules, CA, USA). The membranes were blocked with 5 % nonfat dry milk for 1 h and sequentially probed with anti-collagen II (1:1000; Millipore, Billerica, MA, USA) and anti-β-actin (1:1000; Abcam, Cambridge, MA, USA) primary antibodies. The blots were then incubated with horseradish peroxidase-conjugated secondary antibody (0.1 μg/ml; SantaCruz Biotechnology, Santa Cruz, CA, USA) for 1 h at room temperature. Immunoreactivity bands were detected using the WEST-one western blotting detection system (iNtRON Biotechnology, Seoul, Korea). The bands were quantified using ImageJ software (National Institutes of Health, Bethesda, MD, USA).

For detection of glycosaminoglycan (GAG), WJ-MSCs grown on the culture plate were fixed with 4 % paraformaldehyde (PFA; Yakari Pure Chemicals, Kyoto, Japan) after 14 days. The fixed samples were washed with PBS and stained with 0.5 % Alcian blue (A3157; Sigma-Aldrich, St. Louis, MO, USA) for 30 min for GAG, and rinsed with distilled water. Images were obtained using a light microscope equipped with a video camera, and quantification of the positive staining areas was performed to compare between each group.

### Subcutaneous model in mice

After obtaining Institutional Animal Care and Use Committee (IACUC) approval (No. 130022) prior to using animals, a total of 12 female athymic mice, 7 weeks old (BAL b/c-nude; Orientbio, Seoul, Korea), were divided equally into four groups of three as follows: control group, unoperated group; MSC-lowTR (1 × 10^6^ cells) implanted group; MSC-highTR (1 × 10^6^ cells) implanted group; and composite MSC-highTR/XHA-implanted group. WJ-MSCs and/or XHA were implanted in the dorsal subcutaneous site of the mice. Five weeks after implantation, mice were sacrificed for histological analysis using von Kossa staining (mineralization) and immunostaining for collagen type II.

### Rabbit model of IVD degeneration

Animal experiments were carried out with IACUC approval. IVD degeneration was induced in L3/4, L4/5, and L5/6 IVDs by percutaneous annular puncture and NP aspiration in New Zealand White rabbits, as described previously [14, 27]. Rabbits 4–5 months old and weighing approximately 3.0 kg were used in this study. Briefly, a 21-gauze needle was percutaneously inserted into the center of the IVDs through a posterolateral approach under the C-arm fluoroscopy and the NP was aspirated using a 10 ml syringe. Aspirated disc fragments were carefully examined under dissecting microscopy to confirm that only the NP was aspirated. The mean weight of the NP inside the needle was 7.3 ± 2.2 mg.

### Transplantation of WJ-MSCs in a rabbit model of disc degeneration

At first, a total of 15 rabbits were divided equally into five groups of three to determine the optimal cell dose. The control group was the unoperated group and the sham-operated group underwent induction of disc degeneration without MSC-highTR transplants. After confirmation of IVD degeneration using T2 magnetic resonance imaging (MRI) 3 weeks after induction of IVD degeneration, three groups were given MSC-highTR transplants of 10^5^ (low dose), 10^6^ (middle dose), or 10^7^ (high dose) cells to degenerate L3/4, L4/5, and L5/6 discs using a 25-gauze needle guided by fluoroscopic imaging.

Furthermore, after determining the optimal cell dose, we investigated the effectiveness of expression levels of TβRI/ALK5 and TβRII in WJ-MSCs and combined use of XHA as a cell carrier. Rabbits were thus randomly allocated into four groups of three as follows: the first (XHA) group was given only the XHA scaffold; the second group received only MSC-highTR; in the third group, MSC-lowTR-loaded XHA scaffold was implanted into the NP; and in the fourth group, MSC-highTR-loaded XHA scaffold was implanted. Surgeries for induction of disc degeneration and implantations were performed in a randomized block design. The total volume of injection was always 50 μl.

### MRI of IVDs

T2-weighted MRI was obtained for every animal before experimental use to confirm the absence of abnormalities. Coronal and sagittal T2-weighted MRI scans were obtained in the following setting: time to repetition of 3200 milliseconds, time to echo of 130 milliseconds, 320 (horizontal) × 320 (vertical) matrix; field of view of 120, and 2 mm slices with 0.2 mm spacing between each slice. T2 MRI was performed to confirm IVD degeneration 3 weeks after the induction of IVD degeneration. Following transplantation, serial MRIs were also performed to evaluate disc water content changes at 6 and 12 weeks after transplantation in all groups. The Pfirrmann classification was used for IVD degeneration grading from grade 1 to 5 (1 = normal, 2 = mild degeneration, 3 = moderate degeneration, 4 = severe degeneration, and 5 = advanced degeneration) [28]. All MRI findings were evaluated by two independent spine surgeons.

### Histological analyses

At 12 weeks after the induction of disc degeneration, all rabbits were euthanized with carbon dioxide inhalation and the L1–L6 vertebral bodies from all rabbits were fixed in 4 % PFA. After 2 weeks of decalcification with RapidCal Immuno (BBC Biochemical, Mount Vernon, WA, USA), the tissues were processed individually with paraffin wax embedding. The paraffin blocks were sectioned longitudinally using a microtome into 5 μm thickness. The sections were stained with Masson’s trichrome solution (HT15-1KT, Sigma-Aldrich, St. Louis, MO, USA) for collagen fiber and Alcian blue for GAG, and also immunostained with anti-collagen II antibody. For immunohistochemistry, the sections were washed with PBS, permeabilized for 10 min in 0.25 % Triton X-100 in PBS, and blocked with 1 % BSA in PBS for 30 min to prevent nonspecific binding of proteins. The sections were incubated with monoclonal anti-collagen II antibody (ab34712; Abcam, Cambridge, MA, USA) at 4 °C overnight, and then stained with anti-mouse fluorescein isothiocyanate (FITC)-conjugated secondary antibody (ab8517; Abcam,Cambridge, MA, USA). To determine whether injected human WJ-MSCs could be detected at the end of the study, tissue sections were analyzed using immunohistochemical staining with monoclonal anti-human nucleic antibody (MAB1281; Chemicon, Temecula, CA, USA).

Each sample was interpreted by two independent pathologists. Histological findings included the pattern of the AF, number and nature of cells, amount of matrix, and integrity of the border between the NP and the AF. The findings were quantitatively evaluated with a previously published grading system [29]. The grades were assigned 1–3 points for each of four categories, according to the severity of degeneration. The histological evidence of IVD regeneration was interpreted based on the following criteria: recovery of the lamellar pattern, preservation of the border between the NP and the AF, increased cellularity, and restoration of the ECM. Osteophyte formation and tumor formation were also investigated. To evaluate the regenerated disc, we also morphometrically measured the staining area positive for Masson’s trichrome or Alcian blue staining and calculated the percentages of the positive staining areas with respect to the regenerated disc area.

### Cytokine antibody array

Cytokine secretion by MSC-lowTR and MSC-highTR was examined by a membrane-based antibody array (RayBiotech Inc. Norcross, GA, USA) according to the manufacturer’s instructions. Signal spots were then detected by chemiluminescence.

### Statistical analysis

Three independent experiments for each condition were performed in triplicate. All statistical analyses were performed using SPSS software version 17.0 (SPSS Inc., Chicago, IL, USA). Continuous variables were presented as mean ± standard deviation. Statistical significance was determined between treatment groups for radiological and histological analyses by one-way analysis of variance combined with Tukey’s post-hoc test for comparison between groups. *p* <0.05 was considered statistically significant.

## Results

### Variation in the expression levels of TβRI/ALK5 and TβRII in WJ-MSCs among donors

**Fig. 1:**
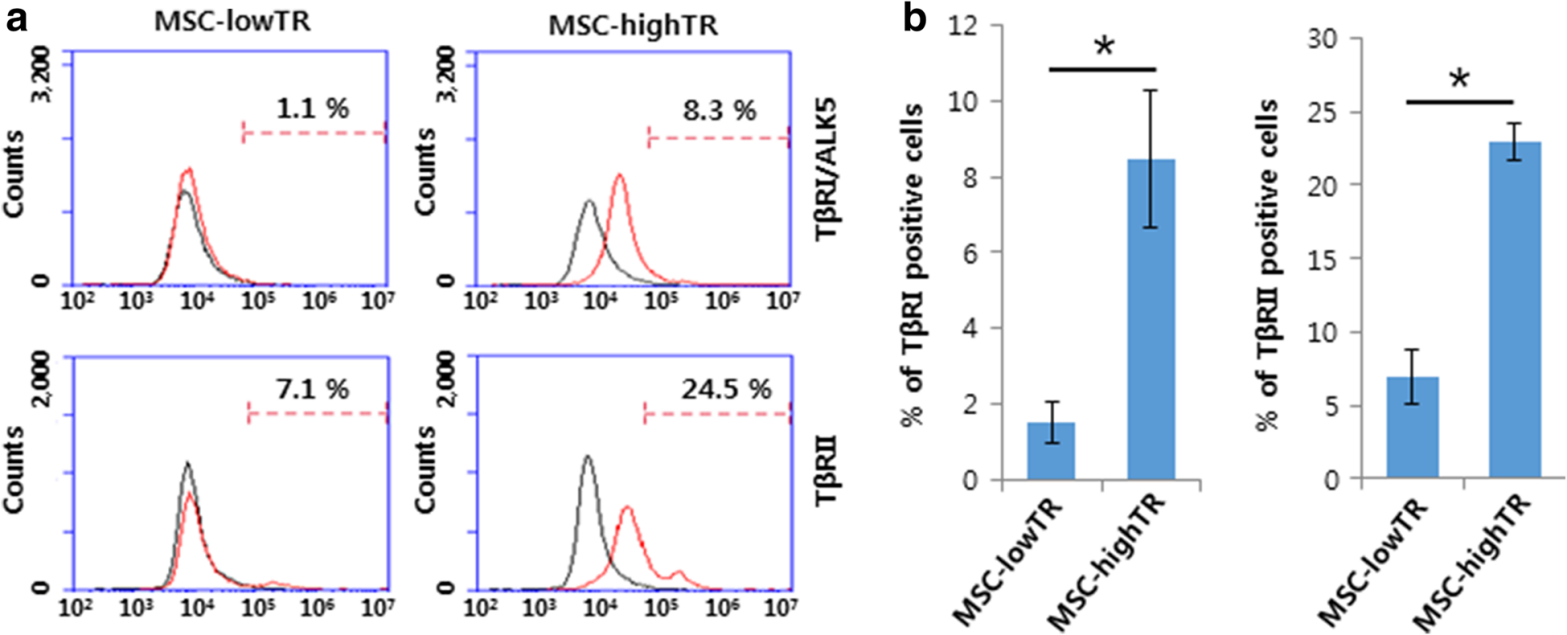
Variation in expression levels of TβRI/ALK5 and TβRII in WJ-MSCs among donors. FACS analysis showing differences in the expression levels of TβRI/ALK5 and TβRII in WJ-MSCs among donors **a**. MSC-highTR are defined as when more than 5.0 % and 20.0 % of cells express TβRI/ALK5 and TβRII, respectively, while MSC-lowTR are defined as when around 1.0 % and 7.0 % of cells express TβRI/ALK5 and TβRII, respectively. **b** Quantification of FACS analysis (*n* = 3). *p <0.05. *ALK5* activin-like kinase receptor 5, *MSC-highTR* Wharton’s Jelly-derived mesenchymal stem cells highly expressing TβRI/ALK5 and TβRII, *MSC-lowTR* Wharton’s Jelly-derived mesenchymal stem cells lowly expressing TβRI/ALK5 and TβRII, *TβR* transforming growth factor-beta receptor

FACS analysis showed that the expression levels of TβRI/ALK5 and TβRII in WJ-MSCs from different donors differed among the donors. Namely, WJ-MSCs from some donors displayed a relatively high percentage of TβRI/ALK5 and TβRII, but WJ-MSCs from others revealed a relatively low percentage of TβRI/ALK5 and TβRII. Based on repeated measurements, MSC-highTR were defined as more than 5.0 % and 20.0 % expression levels of TβRI/ALK5 and TβRII, respectively, while MSC-lowTR were defined when levels were around 1.0 % and 7.0 % respectively (Fig. 1a, b).

### The optimal concentration of XHA for injection of a WJ-MSC-loaded XHA scaffold

A XHA scaffold (Tissuefill®) was used to prevent cell leakage and to enhance the retention of WJ-MSCs at the implantation site. Compared with high concentrations of XHA (1 % and 2 %), droplets of XHA of low concentrations (less than 0.5 %) did not retain their shape and spread out (Figure S1A in Additional file 1). When the culture plate was tilted to an angle of 45°, droplets of XHA at low concentrations (less than 0.5 %) were displaced, whereas droplets of XHA at high concentrations (1 % and 2 %) were not (Figure S1B in Additional file 1). Rheometer analysis revealed that 1 % XHA maintained a uniform viscosity irrespective of the shear rate in comparison with 2 % XHA (Figure S1C in Additional file 1). A 1 % XHA hydrogel solution was thus determined to be suitable for injection of a WJ-MSC-loaded XHA scaffold.

### The viability of WJ-MSCs cultured with a XHA hydrogel

**Fig. 2:**
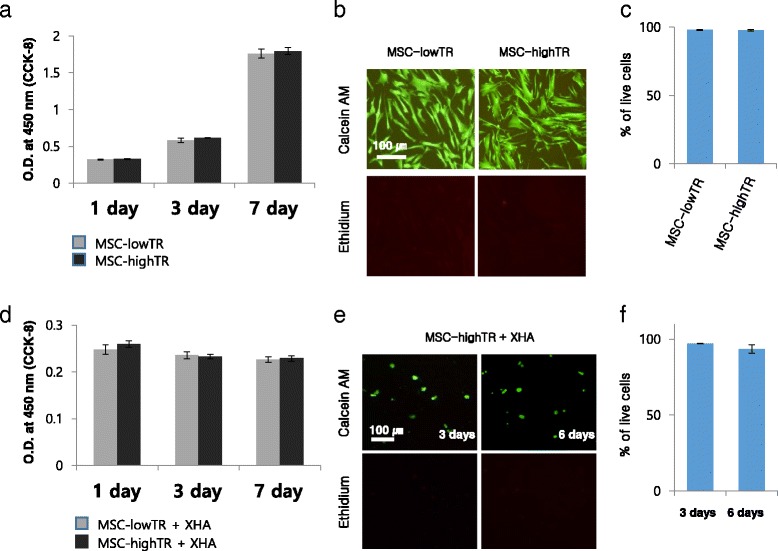
Viability of WJ-MSCs cultured with a XHA hydrogel. **a**–**c** In two-dimensional culture, the CCK-8 and Live/Dead® assays show no difference in cell viability between MSC-highTR and MSC-lowTR, with no cytotoxicity when cells are cultured on a 1 % XHA hydrogel. **d**–**f** The assays show that cell viability is higher than 96 %, even when WJ-MSCs are encapsulated in a 1 % XHA hydrogel. Scale bar, 100 μm (*n* = 3). *ALK5* activin-like kinase receptor 5, *CCK-8* Cell Counting Kit-8, *MSC-highTR* Wharton’s Jelly-derived mesenchymal stem cells highly expressing TβRI/ALK5 and TβRII, *MSC-lowTR* Wharton’s Jelly-derived mesenchymal stem cells lowly expressing TβRI/ALK5 and TβRII, *O.D.* optical density, *TβR* transforming growth factor-beta receptor, *XHA* cross-linked hyaluronic acid

The viability of WJ-MSCs in a 1 % XHA hydrogel was measured using the CCK-8 and Live/Dead® cell viability assays. MSC-highTR and MSC-lowTR were seeded into 1 % XHA and cultured for 1, 3, and 7 days. In two-dimensional culture, these assays revealed no cytotoxicity over 7 days; WJ-MSC viability was higher than 99 % following culture on a 1 % XHA hydrogel for 7 days (Fig. 2a–c). In addition, there was no difference in cell viability between MSC-highTR and MSC-lowTR. Even the viability of WJ-MSCs encapsulated in a 1 % XHA hydrogel was higher than 96 % (Fig. 2d–f). These results suggest that 1 % XHA does not affect the viability of WJ-MSCs in two-dimensional or three-dimensional culture and is suitable for cell delivery.

### Difference in chondrogenic potential between MSC-highTR and MSC-lowTR

**Fig. 3:**
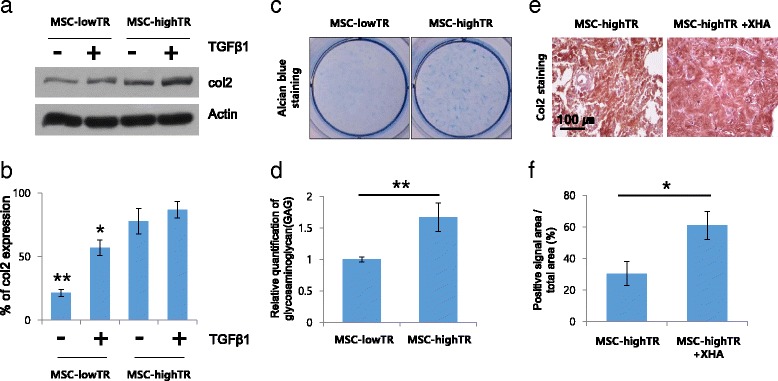
Difference in chondrogenic potential between MSC-highTR and MSC-lowTR. **a** Western blot analysis comparing the expression level of endogenous collagen type II (*col2*) in WJ-MSCs according to their expression levels of TβRI/ALK5 and TβRII as well as TGFβ1 treatment. **b** Quantification of the bands using ImageJ software shows that endogenous collagen type II expression is higher in MSC-highTR than in MSC-lowTR. **c**, **d** Following 2 weeks of culture, Alcian blue staining indicates that MSC-highTR have a much higher chondrogenic differentiation potential than MSC-lowTR. **e**, **f** In mice, immunostaining with an anti-collagen II antibody at 5 weeks after implantation indicates that a MSC-highTR-a loaded XHA scaffold is more effective for chondrogenesis than MSC-highTR alone (*n* = 3). **p <0.01, *p <0.05. *ALK5* activin-like kinase receptor 5, *MSC-highTR* Wharton’s Jelly-derived mesenchymal stem cells highly expressing TβRI/ALK5 and TβRII, *MSC-lowTR* Wharton’s Jelly-derived mesenchymal stem cells lowly expressing TβRI/ALK5 and TβRII, *TβR* transforming growth factor-beta receptor, *TGFβ* transforming growth factor beta, *XHA* cross-linked hyaluronic acid

In vitro, we investigated chondrogenic potential of WJ-MSCs according to the expression levels of TβRI/ALK5 and TβRII and transforming growth factor beta (TGFβ)-1 treatment. WJ-MSCs were cultured as a monolayer in DMEM and were treated with TGFβ1 for 3 days. Analysis of western blot data showed that the expression of endogenous collagen type II was approximately threefold higher in TGFβ1-untreated MSC-highTR (77.8 ± 10.11 %) than in TGFβ1-untreated MSC-lowTR (21.49 ± 2.85 %) based on ImageJ analysis (*p* <0.05). Interestingly, the expression of collagen type II was higher in TGFβ1-treated MSC-highTR (87.03 ± 6.47 %) than in TGFβ1-treated MSC-lowTR (57.05 ± 6.22 %) (Fig. 3a, b). These results suggest that high expression of TβRI/ALK5 and TβRII in WJ-MSCs could facilitate TGFβ1 signal transduction and MSC-highTR may respond well to autocrine TGFβ1 even in the absence of TGFβ1 pretreatment.

Following 2 weeks of culture, Alcian blue staining indicated that MSC-highTR had a much higher chondrogenic differentiation potential than MSC-lowTR (relative quantification of GAG contents stained by Alcian blue: 1.67 ± 0.23 vs. 1.00 ± 0.04; *n* = 3; *p* <0.01) (Fig. 3c, d).

In mice, chondrogenic differentiation was evaluated by immunostaining with an anti-collagen II antibody 5 weeks after subcutaneous implantation. Immunohistochemistry for collagen type II showed that a MSC-highTR-loaded XHA scaffold was more effective for chondrogenesis than MSC-highTR alone (percentage area positively stained by an anti-collagen type II antibody: 60.9 ± 8.9 % vs. 30.46 ± 7.67 %; *n* = 3; *p* <0.05) (Fig. 3e, f). However, mineralization was not observed in any mice which received MSC-highTR alone or MSC-highTR loaded XHA (data not shown). These data suggest that use of a XHA as a cell carrier is also important in inducing cartilage regeneration and that combined use of MSC-highTR and XHA hydrogel can promote IVD regeneration.

### Determination of the optimal number of cells for transplantation in a rabbit model

To determine the optimal cell number for transplantation, we compared the degree of IVD regeneration according to the number of MSC-highTR transplanted. With increased IVD degeneration, Pfirrmann degeneration grading of the discs in T2 MRI increases [28]. T2 MRI of the implanted discs 12 weeks post implantation showed that the Pfirrmann grade was significantly higher in the low-dose group than in the other groups. However, there were no significant differences between the middle and high-dose groups (Additional file 2).

Masson’s trichrome and Alcian blue staining revealed that IVD regeneration was achieved in the middle and high-dose groups, but not in the low-dose group (Figure S3A in Additional file 3). To evaluate regenerated discs, we morphometrically measured the area positively stained by Masson’s trichrome or Alcian blue and calculated the percentages of these positively stained areas with respect to the entire regenerated disc. The percentage areas positively stained by Masson’s trichrome were 14.11 ± 3.64 %, 53.23 ± 6.10 %, and 65.46 ± 5.53 % in the low, middle, and high-dose groups, respectively. The percentage areas positively stained by Alcian blue were 24.29 ± 3.58 %, 48.47 ± 6.06 %, and 58.69 ± 11.82 % in the low, middle, and high-dose groups, respectively (Figure S3B in Additional file 3) (*n* = 3; *p* <0.01). Therefore, histological analyses showed that disc repair in the middle and high-dose groups was superior to that in the low-dose group. The disc structure seemed to be less preserved in the low-dose group, suggesting that 10^5^ WJ-MSCs cannot induce IVD regeneration effectively. There were no significant differences between the middle and high-dose groups. These results suggest that MSC-highTR contribute to disc repair and that 10^6^ WJ-MSCs is the optimal number because fewer cells have shown the least apoptosis with similar regenerative effect compared with a higher injection dose [21, 30, 31]. The scaffold-only group therefore received 50 μl XHA, the cell-only group received WJ-MSCs at a concentration of 10^6^ cells in 50 μl DMEM, and the scaffold with cells groups received a mixture of 25 μl XHA and 25 μl cell suspensions (10^6^ WJ-MSCs in 25 μl DMEM).

### T2-weighted MRI assessment of the restoration of disc water content following transplantation in a rabbit model

**Fig. 4:**
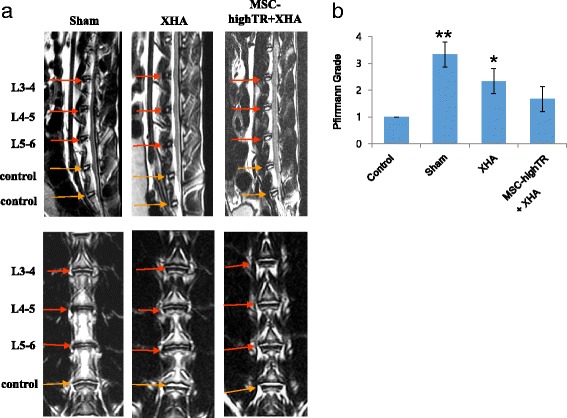
T2-weighted MRI to assess restoration of the disc water content in a rabbit model following transplantation. **a** Representative T2-weighed MRI of the sham, XHA only, and MSC-highTR/XHA groups 12 weeks after transplantation. **b** The Pfirrmann grade is lowest in the MSC-highTR/XHA group, suggesting that combined use of MSC-highTR and a XHA scaffold is most effective for IVD regeneration (*n* = 3). **p <0.01, *p <0.05. *ALK5* activin-like kinase receptor 5, *MSC-highTR* Wharton’s Jelly-derived mesenchymal stem cells highly expressing TβRI/ALK5 and TβRII, *TβR* transforming growth factor-beta receptor, *XHA* cross-linked hyaluronic acid

Three weeks after induction of disc degeneration, a total of 10^6^ WJ-MSCs were transplanted into each degenerated disc and the radiologic and histologic characteristics were analyzed and compared 12 weeks after implantation according to the expression levels of TβRI/ALK5 and TβRII in the transplanted WJ-MSCs and the combined use of a XHA scaffold. T2-weighted MRI performed 12 weeks after transplantation showed that rabbits which received MSC-highTR in a XHA scaffold (MSC-highTR/XHA group) had a Pfirrmann grade of 1.66 ± 0.47, similar to that of control rabbits (1.00 ± 00), twofold lower than that of sham rabbits (3.33 ± 0.47), and 1.4-fold lower than that of rabbits which received XHA only (2.35 ± 0.41) (*p* <0.05 or *p* <0.01 compared with the MSC-highTR/XHA group; Fig. 4a, b). In summary, the Pfirrmann grade of discs, as determined by T2-weighted MRI, was highest in the sham group and was lowest in the MSC-highTR/XHA group, suggesting that the combined use of MSC-highTR and a XHA scaffold is most effective for IVD regeneration.

### Histological analysis of disc regeneration following transplantation in a rabbit model

**Fig. 5:**
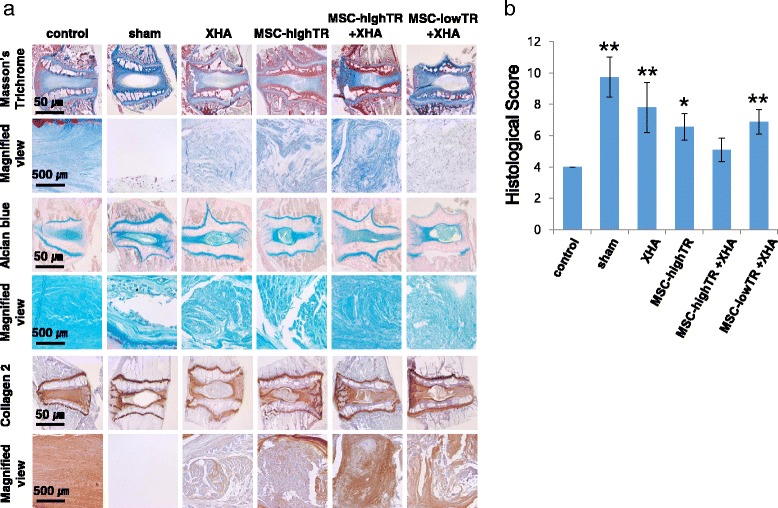
Histological analysis to assess disc regeneration in a rabbit model following transplantation. **a** Comparison of disc regeneration as assessed by histological staining (Masson’s trichrome, Alcian blue staining, and immunostaining with an anti-collagen II antibody). **b** Histological scores to evaluate disc degeneration show that disc regeneration is higher in the MSC-highTR/XHA group than in the other groups. **p <0.01, *p <0.05 compared with the MSC-highTR/HA group. *ALK5* activin-like kinase receptor 5, *MSC-highTR* Wharton’s Jelly-derived mesenchymal stem cells highly expressing TβRI/ALK5 and TβRII, *MSC-lowTR* Wharton’s Jelly-derived mesenchymal stem cells lowly expressing TβRI/ALK5 and TβRII, *TβR* transforming growth factor-beta receptor, *XHA* cross-linked hyaluronic acid

At 12 weeks after transplantation, all harvested discs were studied grossly and histologically. No osteophytes were found in all of the groups and tumors were not detected in any rabbits. The gross appearance of each harvested disc showed more apparent disc space narrowing and connective tissue invasion of the nucleus cavity in the sham-operated group than in the other groups. Histological analysis using Masson’s trichrome staining, Alcian blue staining, and immunostaining for collagen type II showed that the disc structure was well preserved in the MSC-highTR/XHA group in comparison with the other groups (Fig. 5a). With increased IVD degeneration, the histological score increases [29]. Based on a histological scoring system to evaluate IVD degeneration, the histological score was significantly lower in the MSC-highTR/XHA group than in the other treatment groups: control group, 4 points; sham group, 9.72 ± 1.28 points; XHA only group, 7.8 ± 1.6 points; MSC-highTR group, 6.55 ± 0.83 points; MSC-lowTR/XHA group, 6.87 ± 0.78 points; and MSC-highTR/XHA group, 5.08 ± 0.75 points (*p* <0.05 or *p* <0.01 compared with the MSC-highTR/HA group; Fig. 5b). The results thus suggested that combined use of MSC-highTR and a XHA scaffold could provide the highest level of IVD regeneration.

### Fate of implanted cells and a possible mechanism underlying the therapeutic effect

**Fig. 6:**
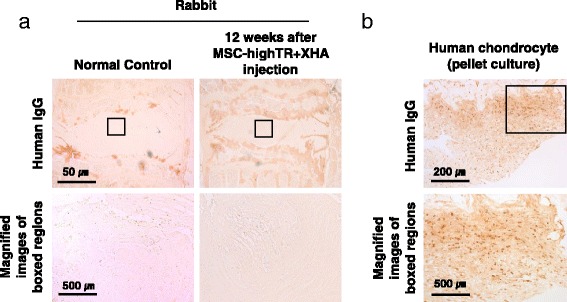
Immunohistochemical characterization of the center of the IVD in a rabbit model following transplantation. **a** Staining with a specific anti-human nucleus antibody shows that cells transplanted into the degenerated disc do not survive at 12 weeks after implantation. **b** Human chondrocytes were used as a positive control. *ALK5* activin-like kinase receptor 5, *IgG* immunoglobulin G, *MSC-highTR* Wharton’s Jelly-derived mesenchymal stem cells highly expressing TβRI/ALK5 and TβRII, *TβR* transforming growth factor-beta receptor, *XHA* cross-linked hyaluronic acid

After confirming that injection of a MSC-highTR-loaded XHA scaffold could promote IVD regeneration, we investigated possible mechanisms underlying this regenerative effect. Transplanted WJ-MSCs were tracked by staining human nuclei. Tissue sections from the MSC-highTR/XHA group did not exhibit immunoreactivity with an anti-human nucleus antibody (Fig. 6). This suggested that the implanted WJ-MSCs do not survive at 12 weeks after transplantation and that paracrine effects of WJ-MSCs injected into the degenerated disc could contribute to IVD regeneration.

**Fig. 7:**
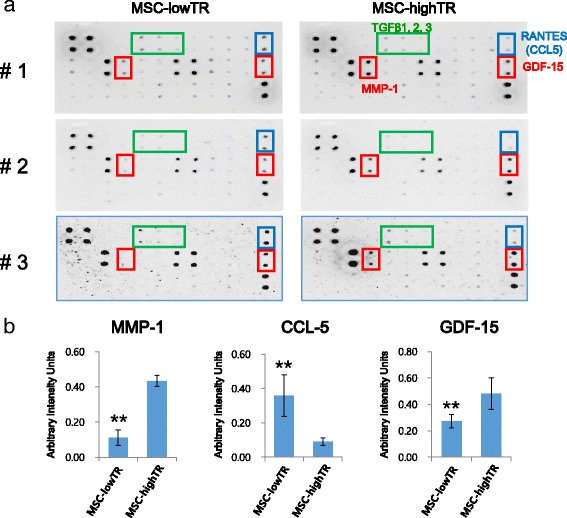
A cytokine antibody array to examine cytokine secretion by WJ-MSCs. **a** A cytokine antibody array was used to compare cytokine secretion between MSC-lowTR and MSC-highTR. TGFβ ligands (TGFβ1, TGFβ2, and TGFβ3) are secreted by both MSC-lowTR and MSC-highTR with no difference. Expression of GDF-15 and MMP-1 is much higher in MSC-highTR than in MSC-lowTR, whereas expression of CCL-5 is much higher in MSC-lowTR than in MSC-highTR. **b** Quantification of the amounts of cytokines secreted by MSC-lowTR and MSC-highTR, as determined by ImageJ analysis (*n* = 3). **p <0.01. *ALK5* activin-like kinase receptor 5, *CCL-5* chemokine (C-C motif) ligand 5, *GDF-15* growth differentiation factor-15, *MMP-1* matrix metalloproteinase-1, *MSC-highTR* Wharton’s Jelly-derived mesenchymal stem cells highly expressing TβRI/ALK5 and TβRII, *MSC-lowTR* Wharton’s Jelly-derived mesenchymal stem cells lowly expressing TβRI/ALK5 and TβRII, *TβR* transforming growth factor-beta receptor

To investigate the difference in the regenerative effect between MSC-lowTR and MSC-highTR, a cytokine antibody array was carried out (Fig. 7). The array revealed that TGFβ ligands (TGFβ1, TGFβ2, and TGFβ3) secreted by both MSC-lowTR and MSC-highTR did not show a big difference. Meanwhile, the expression of growth differentiation factor-15 (GDF-15) and matrix metalloproteinase-1 (MMP-1) was much higher in MSC-highTR than in MSC-lowTR, whereas expression of chemokine (C-C motif) ligand 5 (CCL-5) was much higher in MSC-lowTR than in MSC-highTR. These results suggested that the expression levels of TβRI/ALK5 and TβRII in WJ-MSCs could influence their secretion of cytokines such as GDF-15, MMP-1, and CCL-5 and their response to autocrine TGFβ ligands and that WJ-MSCs could improve IVD degeneration by releasing paracrine factors.

## Discussion

MSCs have been reported to be largely safe and effective for IVD regeneration, and xenograft models have been used to develop novel cell-based therapies for IVD regeneration [12–16]. In this study, we investigated the combined effects of a XHA scaffold and human WJ-MSCs that express TβRI/ALK5 and TβRII differently in a rabbit model of IVD degeneration. The primary findings of this study are as follows: WJ-MSCs from different donors exhibited differential expression of TβRI/ALK5 and TβRII, and their expression levels of these surface receptors affected the degree of IVD regeneration; transplantation of a XHA scaffold with WJ-MSCs resulted in enhanced IVD regeneration compared with transplantation of WJ-MSCs alone; a MSC-highTR-loaded XHA scaffold showed the best regenerative effect; WJ-MSCs did not survive for 12 weeks after transplantation; the therapeutic effects of WJ-MSCs were mediated by the paracrine system; and the regenerative effects of MSC-highTR were mediated, at least in part, by the secretion of MMP-1 and GDF-15.

There was a lack of immune response using in vivo human MSC xenograft models, even though the animals were not treated with immunosuppressive drugs, suggesting an unrecognized immune-privileged site within the IVD space [13, 32]. Compared with other MSCs from bone marrow, adipose tissue, and placenta, WJ-MSCs have the highest proliferative potential, the weakest expression of MHC II genes, and the weakest expression of the immune-related genes including TLR4, TLR3, JAG1, NOTCH2, and NOTCH3 [22–24]. Moreover, a recent study suggested that WJ-MSCs do not require tissue matching; therefore, any donor can give WJ-MSCs to any person without rejection or need for immunosuppressive agents [33]. We did not use immunosuppressive drugs, but the WJ-MSC/XHA-treated discs showed an overall lack of inflammation of the disc. These benefits could make WJ-MSCs an ideal candidate cell for clinical application.

Our data demonstrated that disc levels receiving MSC-highTR/XHA more closely resembled the healthy controls as evidenced in radiological and histological findings. Although there have been several reports on long-term survival of injected MSCs supporting MSC differentiation into disc cells [21, 27], none of the discs revealed no survival of implanted cells at 12 weeks post implantation. This is probably linked with the more severe initial degree of degeneration and the induction of cell death in a harsh environment consisting of low cellularity, low glucose, low oxygen, low pH due to high lactic acid build-up, low nutrients, and an inflammatory milieu [27, 34, 35]. The regenerative effects of MSC-highTR/XHA in our study might therefore be related to immunomodulatory and anti-inflammatory effects via paracrine systems, regardless of whether MSCs differentiate into NP-like cells. In addition, the use of highly expressing TβRI/ALK5 and TβRII and combined use of a XHA scaffold provided the best regenerative effect in vivo.

Inflammatory mediators are a key component to progressive IVD degeneration and the immunomodulatory and immunosuppressive effects of MSCs on NP cells within the degenerated disc may potentially inhibit the inflammatory milieu which could modify the microenvironment of the degenerated disc [36, 37]. WJ-MSCs could be able to function optimally within the inflammatory environment of the degenerated disc to ensure effective repair by the strong immunomodulatory and anti-inflammatory effects [36]. It has also been reported that cytokines secreted by MSCs injected into the degenerated disc could activate endogenous disc cells or endogenous stem cells to induce their migration and the production of other paracrine signals to contribute immediate disc repairing and enhance the regenerative efficiency [12, 36]. Based on our results, TGFβ ligands (TGFβ1, TGFβ2, and TGFβ3) were secreted by both MSC-lowTR and MSC-highTR with no difference, but MSC-highTR could be more responsive to autocrine TGFβ ligands compared with MSC-lowTR. In addition, the secretion of GDF-15 and MMP-1 was much higher in MSC-highTR than in MSC-lowTR, whereas secretion of CCL-5 was much higher in MSC-lowTR than in MSC-highTR. GDF-15 is a secreted protein belonging to the TGFβ superfamily and can induce cartilage formation [38]. MMP-1 is reportedly critical for the mobilization of MSCs toward target tissues because ECM degradation during this migration requires the actions of proteolytic enzymes such as MMP-1 [39]. MMP-1 may play a role in the mobilization of MSCs to the transition zone between the NP and the inner AF region. CCL-5 has been considered to be required for osteogenesis of human MSCs, and decreased expression of CCL-5 in MSC-highTR could provide more effective for IVD regeneration [40]. A complex mixture of cytokines produced by MSC-highTR could thus trigger multiple signaling systems including TGFβ1 signaling and stimulate IVD regeneration.

To prevent cell leakage and reduce the risk of uncontrolled differentiation of MSCs into osteoblasts, the combined use of MSCs and cell carriers, such as fibrin, hyaluronan, and atelocollagen, has been strongly recommended [12, 20, 21, 34, 35, 41, 42]. According to a very recent comprehensive review of 24 animal studies, the rate of complication—particularly osteophyte formation with MSCs—was less than 2.7 % [12]. In the present study, osteophyte formation was not found in any groups because the XHA scaffold helps prevent cell leakage, promote cell attachment, exert an anti-inflammatory effect, and provide a favorable microenvironment for IVD regeneration [43].

Our study showed no evidence that injected WJ-MSCs could differentiate into NP-like cells. NP cells and matrix components in disc tissue have been suggested to have positive modulatory effects on MSC growth and to show differentiation towards an NP-like phenotype in vivo [36], and MSCs could be differentiated into NP-like cells by preconditioning MSCs with growth factors such as TGFβ and bone morphogenetic proteins in vitro [44], culture under low oxygen prior to implantation [45], coculture of MSCs with NP cells [46, 47], or notochordal cell-conditioned media [48]. However, there is currently a lack of collective evidence to support that injected MSCs only differentiate into NP cells inside the degenerated disc because outcome is more difficult to interpret by the other assessment methods, such as by quantitative reverse transcription PCR (RT-qPCR), due to lack of well-defined NP versus chondrocyte markers [12, 36]. The outcome of most studies thus appears to be valid based on T2-weighted MRI and histological grading.

## Conclusions

This is the first study to address the efficacy of WJ-MSCs for the treatment of disc degeneration according to the expression levels of TβRI/ALK5 and TβRII in a rabbit model. Compared with MSC-lowTR, MSC-highTR exhibited a higher susceptibility to TGFβ1 and secreted paracrine cytokines for IVD regeneration, enhancing their regenerative effect by immediate and trophic effects of from WJ-MSCs. Furthermore, combined use of MSC-highTR and a XHA scaffold enhanced IVD regeneration due to its probable effects, including promotion of cell attachment, anti-inflammatory effect, or provision of a microenvironment that is more suitable for the NP. This strategy has an advantage in terms of future clinical applications because enhancement of the regenerative power of MSCs can be achieved with no potentially harmful modifications of MSCs.

## Additional files

Additional file 1: Figure S1. Showing determination of the optimal concentration of XHA. A Droplets of XHA at low concentrations (0.1 % and 0.5 %) do not hold their shape and spread out. B When the culture plate is tilted to an angle of 45°, droplets of XHA at high concentrations (1 % and 2 %) are not displaced. C Rheometer analysis shows that 1 % XHA maintains a constant viscosity regardless of the shear rate. Additional file 2: Figure S2. Showing MRI to determine the optimal number of WJ-MSCs required for disc regeneration in a rabbit model. A Representative images of sagittal and coronal T2-weighted magnetic resonance images at 12 weeks after the transplantation of various numbers of cells. B The Pfirrmann grade was determined to establish the degree of degeneration. Degeneration is more severe in the low-dose group than in the middle and high-dose groups. However, there are no significant differences between the middle and high-dose groups (*n* = 3). ***p* <0.01; **p* <0.05. Additional file 3: Figure S3. Showing histological analysis to determine the optimal number of WJ-MSCs required for disc regeneration in a rabbit model. A Comparison of Masson’s trichrome and Alcian blue staining according to the number of cells transplanted. B Quantification of the percentage area positively stained by Masson’s trichrome or Alcian blue. Disc repair in the middle and high-dose groups is superior to that in the low-dose group, and there is no significant difference between the middle and high-dose groups. This suggests that 10^6^ WJ-MSCs is the minimum number of cells required for effective disc regeneration in a rabbit model of disc degeneration (*n* = 3). ***p* <0.01.

## Abbreviations

AD-MSC: Adipose tissue-derived mesenchymal stem cell
AF: Annulus fibrosus
ALK5: Activin-like kinase receptor 5
BSA: Bovine serum albumin
CCK-8: Cell Counting Kit-8
CCL-5: Chemokine (C-C motif) ligand 5
DMEM: Dulbecco’s modified Eagle’s medium
DMEM-L: Dulbecco’s modified Eagle’s medium with low glucose
ECM: Extracellular matrix
FACS: Fluorescence-activated cell sorting
FBS: Fetal bovine serum
FITC: Fluorescein isothiocyanate
GAG: Glycosaminoglycan
GDF-15: Growth differentiation factor-15
GMP: Good Manufacturing Practice
IACUC: Institutional Animal Care and Use Committee
IVD: Intervertebral disc
MMP-1: Matrix metalloproteinase-1
MRI: Magnetic resonance imaging
MSC-highTR: Wharton’s Jelly-derived mesenchymal stem cells highly expressing TβRI/ALK5 and TβRII
MSC-lowTR: Wharton’s Jelly-derived mesenchymal stem cells lowly expressing TβRI/ALK5 and TβRII
MSC: Mesenchymal stem cell
NP: Nucleus pulposus
PBS: Phosphate-buffered saline
PFA: 4 % paraformaldehyde
PVDF: polyvinylidene fluoride
RT-qPCR: quantitative reverse transcription PCR
TGFβ: Transforming growth factor beta
TβR: Transforming growth factor-beta receptor
WJ-MSC: Wharton’s Jelly-derived mesenchymal stem cell
XHA: Cross-linked hyaluronic acid
