# Supplementary material for: Transplantation of human Wharton’s jelly-derived mesenchymal stem cells highly expressing TGFβ receptors in a rabbit model of disc degeneration
Source: Stem Cell Res Ther. 2015 Oct 2;6:190. doi: 10.1186/s13287-015-0183-1 (PMC4592544; doi:10.1186/s13287-015-0183-1)
Supplement: Additional file 1: Figure S1. — Showing determination of the optimal concentration of XHA. A Droplets of XHA at low concentrations (0.1 % and 0.5 %) do not hold their shape and spread out. B When the culture plate is tilted to an angle of 45°, droplets of XHA at high concentrations (1 % and 2 %) are not displaced. C Rheometer analysis shows that 1 % XHA maintains a constant viscosity regardless of the shear rate. [file 13287_2015_183_MOESM1_ESM.pptx]

## Slide 1
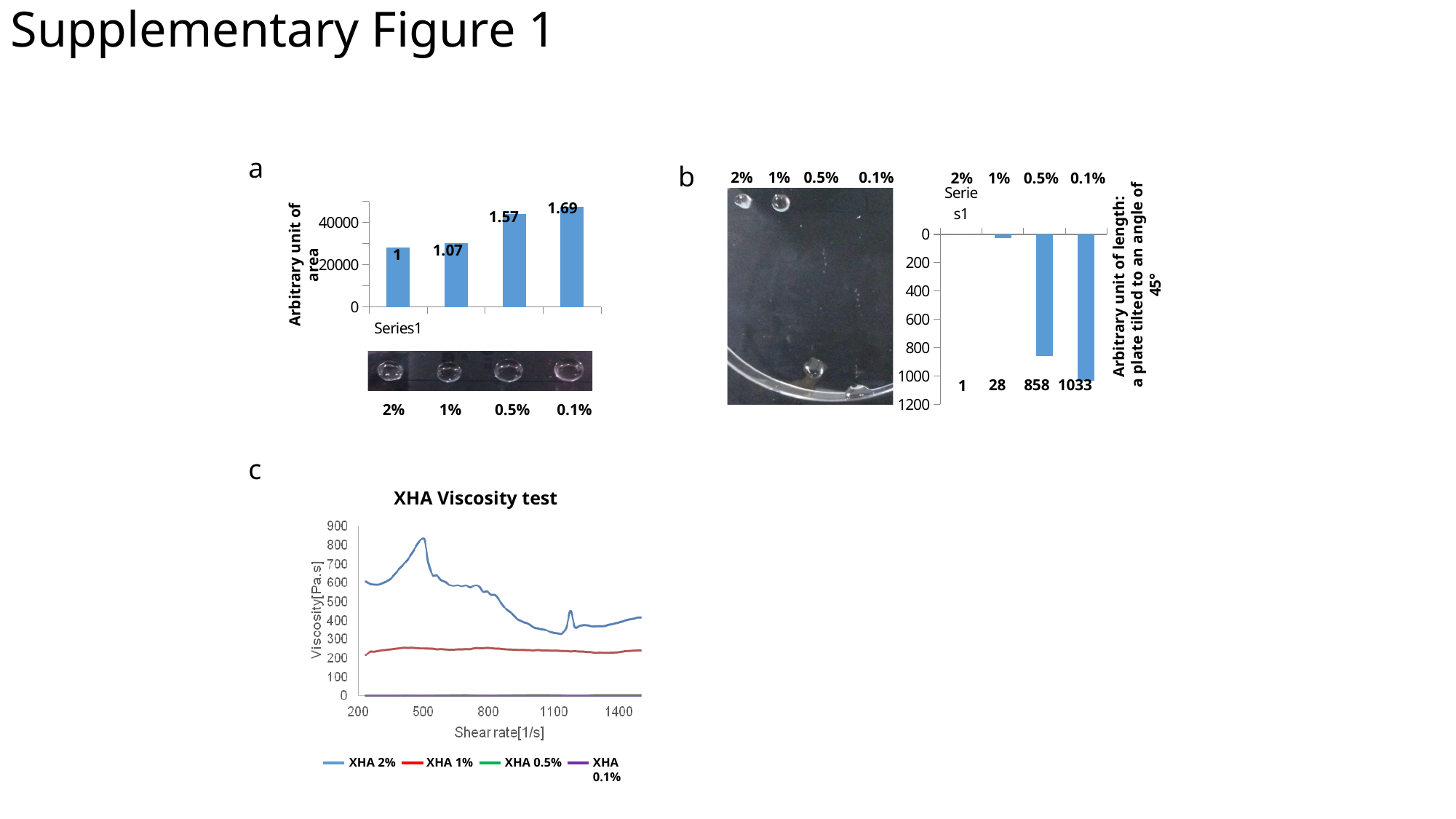

Supplementary Figure 1
a
b
2%
1%
0.5%
0.1%
2%
1%
0.5%
0.1%
### Chart
| Category | |
|---|---|
| | 0.0 |
| | 28.0 |
| | 858.0 |
| | 1033.0 |
Arbitrary unit of length:
a plate tilted to an angle of 45°
28
858
1033
1
1.69
### Chart
| Category | |
|---|---|
| | 28041.0 |
| | 30139.0 |
| | 43922.0 |
| | 47483.0 |1.57
1.07
1
Arbitrary unit of area
2%
1%
0.5%
0.1%
c
XHA Viscosity test
XHA 2%
XHA 1%
XHA 0.5%
XHA 0.1%
