# Supplementary material for: Transplantation of human Wharton’s jelly-derived mesenchymal stem cells highly expressing TGFβ receptors in a rabbit model of disc degeneration
Source: Stem Cell Res Ther. 2015 Oct 2;6:190. doi: 10.1186/s13287-015-0183-1 (PMC4592544; doi:10.1186/s13287-015-0183-1)
Supplement: Additional file 2: Figure S2. — Showing MRI to determine the optimal number of WJ-MSCs required for disc regeneration in a rabbit model. A Representative images of sagittal and coronal T2-weighted magnetic resonance images at 12 weeks after the transplantation of various numbers of cells. B The Pfirrmann grade was determined to establish the degree of degeneration. Degeneration is more severe in the low-dose group than in the middle and high-dose groups. However, there are no significant differences between the middle and high-dose groups (n = 3). **p <0.01; *p <0.05. [file 13287_2015_183_MOESM2_ESM.pptx]

## Slide 1
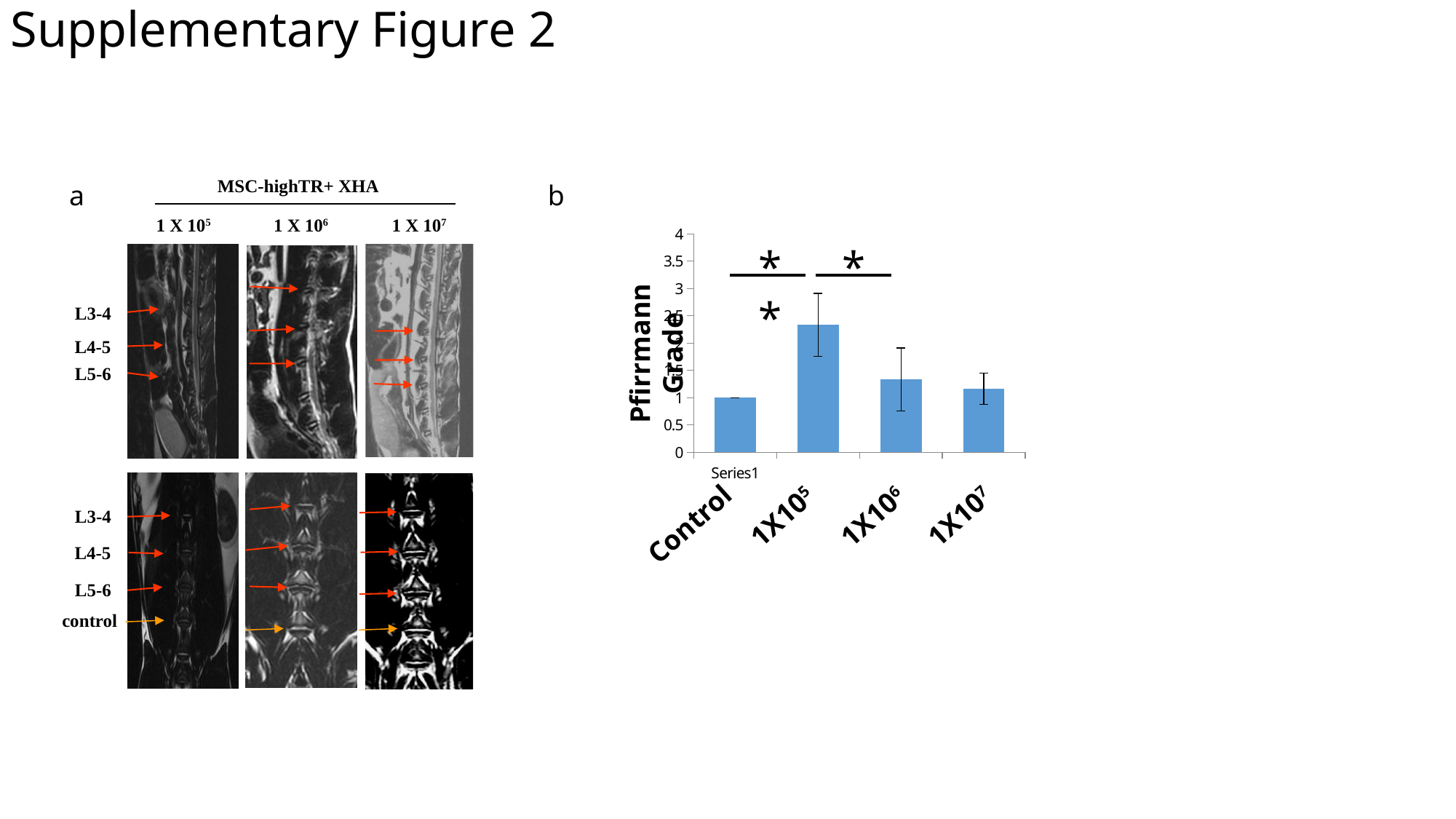

Supplementary Figure 2
MSC-highTR+ XHA
a
b
1 X 105
1 X 106
1 X 107
### Chart
| Category | |
|---|---|
| | 1.0 |
| | 2.3333333333333335 |
| | 1.3333333333333333 |
| | 1.1666666666666667 |Pfirrmann Grade
1X105
1X106
1X107
Control
**
*
L3-4
L4-5
L5-6
L3-4
L4-5
L5-6
control
