# Supplementary material for: Transplantation of human Wharton’s jelly-derived mesenchymal stem cells highly expressing TGFβ receptors in a rabbit model of disc degeneration
Source: Stem Cell Res Ther. 2015 Oct 2;6:190. doi: 10.1186/s13287-015-0183-1 (PMC4592544; doi:10.1186/s13287-015-0183-1)
Supplement: Additional file 3: Figure S3. — Showing histological analysis to determine the optimal number of WJ-MSCs required for disc regeneration in a rabbit model. A Comparison of Masson’s trichrome and Alcian blue staining according to the number of cells transplanted. B Quantification of the percentage area positively stained by Masson’s trichrome or Alcian blue. Disc repair in the middle and high-dose groups is superior to that in the low-dose group, and there is no significant difference between the middle and high-dose groups. This suggests that 106 WJ-MSCs is the minimum number of cells required for effective disc regeneration in a rabbit model of disc degeneration (n = 3). **p <0.01. [file 13287_2015_183_MOESM3_ESM.pptx]

## Slide 1
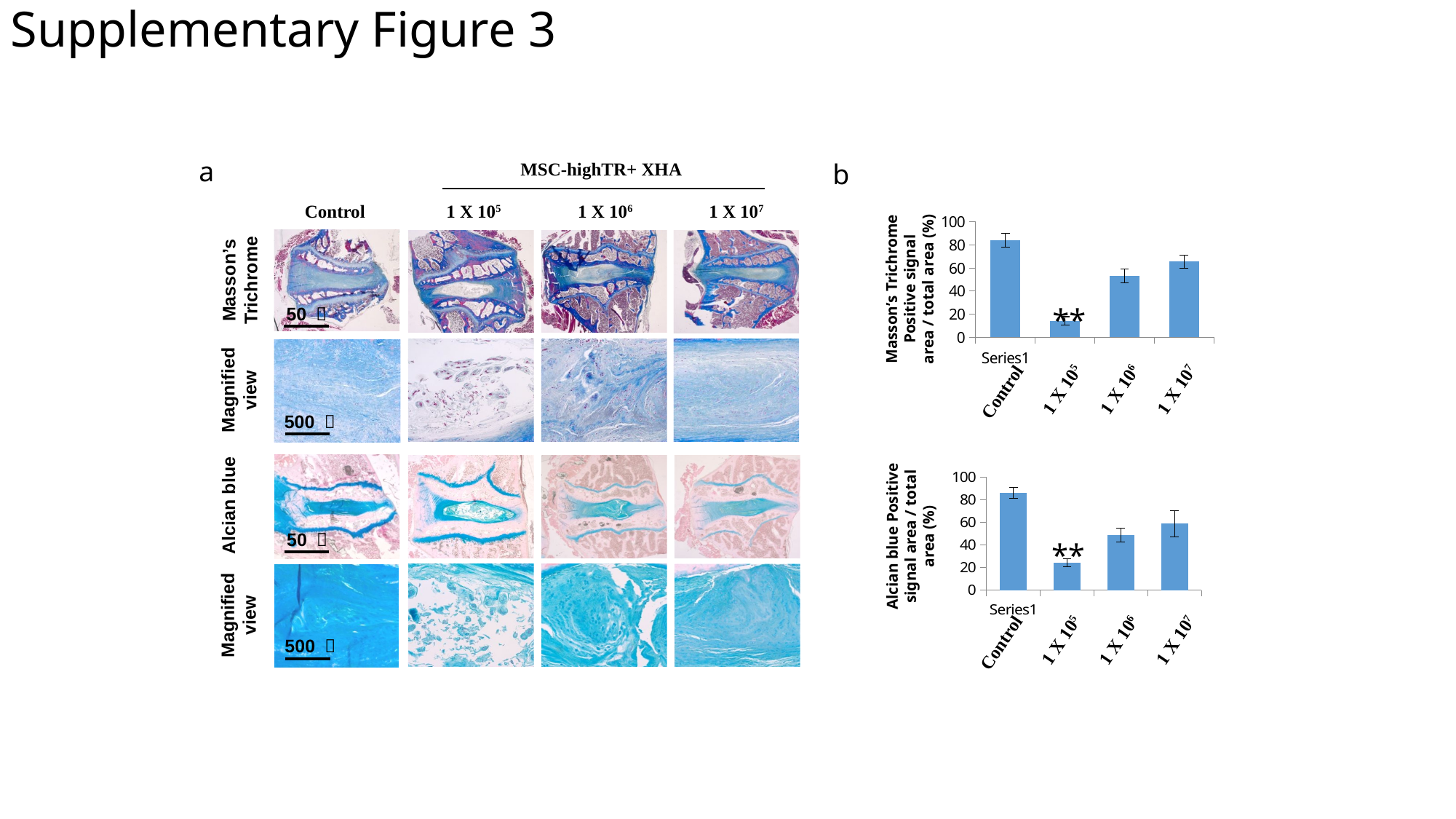

Supplementary Figure 3
a
b
MSC-highTR+ XHA
1 X 105
1 X 106
1 X 107
Control
### Chart
| Category | |
|---|---|
| | 83.91533333333331 |
| | 14.115333333333334 |
| | 53.23200000000006 |
| | 65.46566666666669 |
Masson’s Trichrome
Masson’s Trichrome Positive signal area / total area (%)
**
50 ㎛
Magnified view
1 X 105
1 X 106
1 X 107
Control
500 ㎛
### Chart
| Category | |
|---|---|
| | 86.06533333333319 |
| | 24.290666666666667 |
| | 48.47266666666648 |
| | 58.692000000000085 |Alcian blue Positive signal area / total area (%)
Alcian blue
50 ㎛
**
Magnified view
1 X 105
1 X 106
1 X 107
Control
500 ㎛
